# Harmonizing Formula Prescription Patterns in Patients With Chronic Kidney Disease: A Population-Based Cross-Sectional Study

**DOI:** 10.3389/fphar.2021.573145

**Published:** 2021-04-29

**Authors:** Hung-Lung Lin, Ming-Yen Lin, Cheng-Hsun Tsai, Yi-Hsiu Wang, Chung-Jen Chen, Shang-Jyh Hwang, Ming-Hong Yen, Yi-Wen Chiu

**Affiliations:** ^1^ Department of Chinese Medicine, Kaohsiung Medical University Hospital, Kaohsiung Medical University, Kaohsiung, Taiwan; ^2^ Division of Nephrology, Department of Internal Medicine, Kaohsiung Medical University Hospital, Kaohsiung Medical University, Kaohsiung, Taiwan; ^3^ Master of Public Health Degree Program, College of Public Health, National Taiwan University, Taipei, Taiwan; ^4^ Department of Renal Care, College of Medicine, Kaohsiung Medical University, Kaohsiung, Taiwan; ^5^ Graduate Institute of Medicine, College of Medicine, Kaohsiung Medical University, Kaohsiung, Taiwan; ^6^ Institute of Population Health Sciences, National Health Research Institutes, Miaoli, Taiwan; ^7^ School of Pharmacy, College of Pharmacy, Kaohsiung Medical University, Kaohsiung, Taiwan

**Keywords:** chronic kidney disease, Chinese herbal medicine, traditional Chinese medicine, Chinese herbal formula, prescription patterns

## Abstract

**Objective:** Harmonizing formulas are associated with beneficial renal outcomes in chronic kidney disease (CKD), but the therapeutic mechanisms are unclear. The study aims to explore the associations of intentions and independent factors with harmonizing formulas prescriptions for patients with CKD.

**Methods:** We conducted a population-based cross-sectional study to explore factors associated with harmonizing formulas prescription. Patients who had been prescribed harmonizing formulas after CKD diagnosis was defined as the using harmonizing formulas group. Disease diagnoses when having harmonizing formula prescriptions and patient characteristics related to these prescriptions were collected.

**Results:** In total, 24,971 patients were enrolled in this analysis, and 5,237 (21%) patients were prescribed harmonizing formulas after CKD diagnosis. The three most frequent systematic diseases and related health problems for which harmonizing formula prescriptions were issued in CKD were symptoms, signs, and ill-defined conditions (24.5%), diseases of the digestive system (20.67%), and diseases of the musculoskeletal system (12.9%). Higher likelihoods of harmonizing formula prescriptions were associated with young age (adjusted odds ratio: 0.98, 95% confidence interval: 0.97–0.98), female sex (1.79, 1.68–1.91), no diabetes (1.20, 1.06–1.36), no hypertension (1.38, 1.27–1.50), no cerebrovascular disease (1.34, 1.14–1.56), less disease severity (0.85, 0.83–0.88), using nonsteroidal anti-inflammatory drugs (NSAIDs) (1.65, 1.54–1.78), and using analgesic drugs other than NSAIDs (1.47, 1.35–1.59).

**Conclusion:** Harmonizing formulas are commonly used for treating symptoms of the digestive and musculoskeletal systems in CKD cases. Further research on harmonizing formula effectiveness with regard to particular characteristics of CKD patients is warranted.

## Introduction

Chronic kidney disease (CKD), defined as substantial renal damage persisting for three months, is prevalent and affects 8–16% of adults globally (Jha et al., 2013). CKD can be regarded as an irreversible condition. After a patient develops CKD, the renal function might progressively deteriorate over several years until reaching end-stage renal disease (ESRD) (Zhong et al., 2017). Although guidelines have recommended that CKD should be appropriately managed to slow its progression (Levey et al., 2002; Levin et al., 2013), maintaining stable renal function and relieving the symptoms and signs caused by accumulation of uremic toxins remain challenging. Chinese herbal medicines (CHMs), through syndrome differentiation and treatment selection, present a means to improve CKD care and may stabilize renal function.

Improving syndromes by treatment is the main philosophy of CHMs (Jiang et al., 2012). Syndromes could be differentiated and summarized by integrating four main comprehensive evaluations of symptoms through observation, listening, questioning, and pulse analyses (Chan, 1995). After differentiating syndromes from one patient, a physician would prescribe natural products following the principle of chief-deputy-assistant-envoy. The chief herb product within one prescription provides the main therapeutic effect for syndromes (Su et al., 2016).

Several experimental animal studies have concluded that certain herbs (e.g., *Rheum officinale Balli.*, Rhubarb) in CHM have potential anti-inflammatory and antifibrotic effects and could be an agent in renal fibrosis therapy (Lu et al., 2015; Zhao et al., 2016). However, these findings are less applicable in clinical practice because illnesses in humans are often more complex than those in mice. For example, CKD in humans may have different etiologies and be accompanied by noncommunicable diseases. Therefore, observational human studies of CHM treatment effects provide an opportunity to understand current practices in CHM therapy and help explore new therapeutic formulas for use in CKD. Studies and our previous data have demonstrated the potential beneficial effects of CHM on CKD outcomes (Hsieh et al., 2014; Hsu et al., 2014; Lin et al., 2015; Hsieh et al., 2017), highlighting that further understanding of the target population in CKD for treatment with specific formulas is warranted.

Our previous study identified harmonizing formulas as having the most potential benefits for renal progression in CHMs. Usually, they are commonly used to treat disorders caused by contrasting illnesses including shaoyang (half interior and half exterior) syndromes, liver and spleen disharmonies, and intestine and stomach disharmonies (Chen and Chen, 2009). The effectiveness of harmonizing formulas has been widely recognized in reducing depressive syndromes (Yeung et al., 2015; Zeng et al., 2017) and improving survival in patients with stroke and cancer (Liao et al., 2015). However, applications for patients with CKD remain relatively less understood. Our study explored disease categories for which harmonizing formulas were prescribed and independent factors associated with harmonizing formula prescriptions in patients with CKD.

## Methods

### Data Source and Subjects

The Taiwan government launched the National Health Insurance (NHI) program in 1995. It covers 99.6% of Taiwan’s population and has service contracts with 93% of health care institutes. NHI reimburses medications (Western medicine and CHM), ambulatory, emergency, dental, and inpatient care after health care institutes complete medical services and uploads claims data. The claims data are further managed by the Taiwan National Health Research Institutes for inclusion in the National Health Insurance Research Database (NHIRD) and are available for academic research.

We conducted a population-based cross-sectional study by using the Longitudinal Health Insurance Database 2000 (LHID2000), a subset of the NHIRD. The LHID2000 contains the data of 1 million randomly sampled patients who were NHI beneficiaries in 2000. The randomly sampled patients exhibit similar distributions in age, sex, birth year, and average insured payroll-related amount with the general population. This research was approved by the Institutional Review Board of Kaohsiung Medical University Hospital (KMUHIRB-EXEMPT(I)-20150063). All research procedures followed the guidelines of the Declaration of Helsinki.

### CKD

The detailed methods of this study were in our previous study (Lin et al., 2015). In brief, we identified patients who received new diagnoses of CKD between 2000 and 2005 by using the frequency of appearance of specific *International Classification of Diseases, Ninth Revision, Clinical Modification* (ICD-9-CM) codes within 1 year (Collins et al., 2009). Although laboratory data were lacking in these databases, we can reasonably assume that most of these patients had stage 3–5 CKD [estimated glomerular filtration rate (eGFR) < 60 ml/min/1.73 m^2^] on the basis of regional hospital data using the same algorithm. We excluded patients who were aged <18 or ≥85 years (*n* = 2, 673), had cancer (*n* = 1,680) or underwent dialysis before receiving a CKD diagnosis (*n* = 28), or had received any CHM prescription within 1 year before diagnosis (*n* = 11,104). Therefore, the cohort was suitable to assess for determining factors associated with CHM prescription.

### Harmonizing Formulas

All CHM prescriptions from CKD diagnosis to the start of dialysis or the end of 2008 were collected. We distinguished these CHM prescriptions into harmonizing formulas and other formulas according to approaches suggested in textbooks, with minor modifications (Cao, 2000; Chen and Chen, 2009). To detect potential indicators for using harmonizing formulas, disease categories for prescribing harmonizing and other formulas were compared on the basis of a patient’s first ICD-9-CM diagnosis code of the prescription and classified into various disease system and health problem groups. These systematic diseases and related health problems can be divided into infections and parasitic diseases (ICD-9-CM: 001–139); neoplasms (140–239); endocrine, nutritional, and metabolic diseases, and immunity disorders (240–279); diseases of the blood and blood-forming organs (280–289); mental disorders (290–319); diseases of the nervous system and sensory organs (320–389); diseases of the circulatory system (390–459); diseases of the respiratory system (460–519); diseases of the digestive system (520–579); diseases of the genitourinary system (580–629); complications of pregnancy, childbirth, and the puerperium (630–679); diseases of the skin and subcutaneous tissue (680–709); diseases of the musculoskeletal system (710–739); congenital anomalies (740–759); certain conditions originating in the perinatal period (760–779); symptoms, signs, and ill-defined conditions (780–799); injury and poisoning (800–999); and supplementary classification and others (V01–V82, E800–E999).

### Assessment of Associated Factors

We collected information on patient characteristics, namely age, sex (male or female), insurance amount (fixed premium or dependent, <New Taiwan dollar (NT$)20,000, NT$20,000–39,999, and ≥NT$39,999 [∼US$1,333]), region (north, center, south, and east), urbanization of residence (urban or rural), main comorbidities (acute coronary syndrome, diabetes, hypertension, hyperlipidemia, chronic obstructive pulmonary disease (COPD), and cerebrovascular disease), Charlson comorbidity index score, and primary Western medicines used (diabetic drugs, antihypertensive drugs, nonsteroidal anti-inflammatory drugs (NSAIDs), analgesic drugs other than NSAIDs, and antilipid drugs) during the observation period.

### Statistical Analysis

Continuous and categorical data were expressed as mean ± standard deviation or median (interquartile range) and percentage, respectively. Significant differences in patient characteristics between the harmonizing formula use and nonuse groups were evaluated using an independent *t* test for continuous variables and *χ* ^2^ test for categorical variables. We used the proportion of disease categories to determine the differences in disease treatment between using harmonizing formulas and other formulas. In addition, a multivariable logistic regression with backward elimination procedure (*α* = 0.2) of all collected factors was performed to identify the independent factors of harmonizing formula prescription. Results of the logistic regression were represented as odds ratios (O.R.s) and 95% confidence intervals (C.I.s). All statistical operations were performed in SAS (version 9.4, SAS Institute, Cary, NC, United States). A *p* value < 0.05 was considered significant.

## Results

### Patient Characteristics by Use of Harmonizing Formulas

We included 24,971 patients who received new diagnoses of CKD, and 21% of these patients were prescribed harmonizing formulas. Compared with patients in the harmonizing formulas nonuse group, patients in the group using harmonizing formulas were significantly more likely to be young, female, and living in central Taiwan and urban areas. They were significantly more likely to have high insurance amounts, fewer comorbidities (acute coronary syndrome, diabetes, hypertension, hyperlipidemia, COPD, or cerebrovascular disease), less disease severity, and less use of diabetic and antihypertensive drugs, but more use of NSAIDs, analgesic drugs other than NSAIDs, and anti-lipid drugs (Table 1).

**TABLE 1:** Characteristics of study cohort by harmonizing formula use.

	Harmonizing formulas		*p* value
Characteristic	Nonuse	Use	
Patient no	19,734	5,237	
Age, y	58.3 ± 16.4	51.3 ± 15.6	<0.001
Female (%)	37.9	50.2	<0.001
Insurance amount, NT$ (%)			<0.001
Fixed premium or dependent	20.5	18.8	
<20,000	58.1	56.4	
20,000–39,999	13.7	16.4	
≧39,999	7.7	8.5	
Region (%)			<0.001
North	46.6	43.9	
Center	21.1	23.6	
South	28.5	29.7	
East	3.7	2.8	
Urbanization (%)			<0.001
Urban	72.9	76.6	
Comorbidities (%)			
Acute coronary syndrome	12.5	7.7	<0.001
Diabetes	29.9	19.4	<0.001
Hypertension	40.7	26.6	<0.001
Hyperlipidemia	13.5	11.1	<0.001
COPD	9.2	5.4	<0.001
Cerebrovascular disease	11.5	4.3	<0.001
Charlson score			
Mean ± SD	1.49 ± 1.87	0.82 ± 1.28	<0.001
Median (IQR)	1 (0–2)	0 (0–1)	<0.001
Confounding drugs, %			
Diabetic drugs	29.7	24.6	<0.001
Antihypertensive drugs	55.6	51.1	<0.001
NSAIDs	29.4	40.6	<0.001
Analgesic drugs other than NSAIDs	42.9	45.8	<0.001
Anti-lipid drugs	20.9	21.1	<0.001

Abbreviations: COPD, chronic obstructive pulmonary disease; IQR, interquartile range; NSAIDs, nonsteroidal anti-inflammatory drugs; NT$, New Taiwan Dollar.

The differences in characteristics among groups were compared using *χ* ^2^ tests for categorical variables and independent *t* tests for continuous variables. A *p* value of <0.05 was considered statistically significant.

NT$30 equals approximately US$1.

### Disease Categories for Prescribing Harmonizing Formulas

The disease categories for prescribing harmonizing formulas and the other types of Chinese herbal formulas in patients with CKD were compared and are listed in Table 2. The three most frequent disease categories for prescribing harmonizing formula in CKD were symptoms, signs, and ill-defined conditions (24.5%); diseases of the digestive system (20.67%); and diseases of the musculoskeletal system (12.9%). Similar frequencies of disease categories were observed for other formula prescriptions in patients with CKD. Notably, an increased proportion of patients with diseases of the digestive system (6.39% difference) and a reduced proportion of those with diseases of the respiratory system (−5.5% difference) received harmonizing formula prescriptions compared with other formula prescriptions in this analysis.

**TABLE 2:** Systematic diseases and related health problems for harmonizing formula prescription claims.

Disease category (ICD-9-CM)	Harmonizing formulas (a)	Other formulas (b)	Difference [(a) − (b)]	Ratio [(a)/(b)]
Infection and parasitic diseases (001–139)	0.87	0.52	0.35	1.67
Neoplasms (140–239)	0.78	0.68	0.10	1.15
Endocrine, nutritional, and metabolic diseases and immunity disorders (240–279)	3.52	5.02	−1.5	0.7
Diseases of the blood and blood-forming organs (280–289)	0.32	0.37	−0.05	0.86
Mental disorders (290–319)	1.76	0.9	0.86	1.96
Diseases of the nervous system and sense organs (320–389)	3.37	3.71	−0.34	0.91
Diseases of the circulatory system (390–459)	2.49	4.29	−1.80	0.58
Diseases of the respiratory system (460–519)	6.54	12.09	−5.55	0.54
Diseases of the digestive system (520–579)	20.67	14.28	6.39	1.45
Diseases of the genitourinary system (580–629)	12.38	11.93	0.45	1.04
Complications of pregnancy, childbirth, and the puerperium (630–679)	0.17	0.18	−0.01	0.94
Diseases of the skin and subcutaneous tissue (680–709)	2.10	2.90	−0.80	0.72
Diseases of the musculoskeletal system (710–739)	12.9	12.51	0.39	1.03
Congenital anomalies (740–759)	0.37	0.28	0.09	1.32
Certain conditions originating in the perinatal period (760–779)	0.01	0.00	0.01	-
Symptoms, signs, and ill-defined conditions (780–799)	24.50	24.54	−0.04	1.00
Injury and poisoning (800–999)	5.48	3.51	1.97	1.56
Supplementary classification and others (V01–V82, E800–E999)	1.78	2.27	−0.49	0.78

Abbreviations: ICD-9-CM, International Classification of Diseases, Ninth Revision, Clinical Modification; aOR, adjusted odds ratio; CI, confidence interval.

Number of Chinese medicine visits is 24,226 for harmonizing formulas and 622,790 for other formulas.

Proportion of disease categories by harmonizing and other formulas are displayed.

### Prescription Frequency of Constituent Herbs in Harmonizing Formulas

**FIGURE 1:**
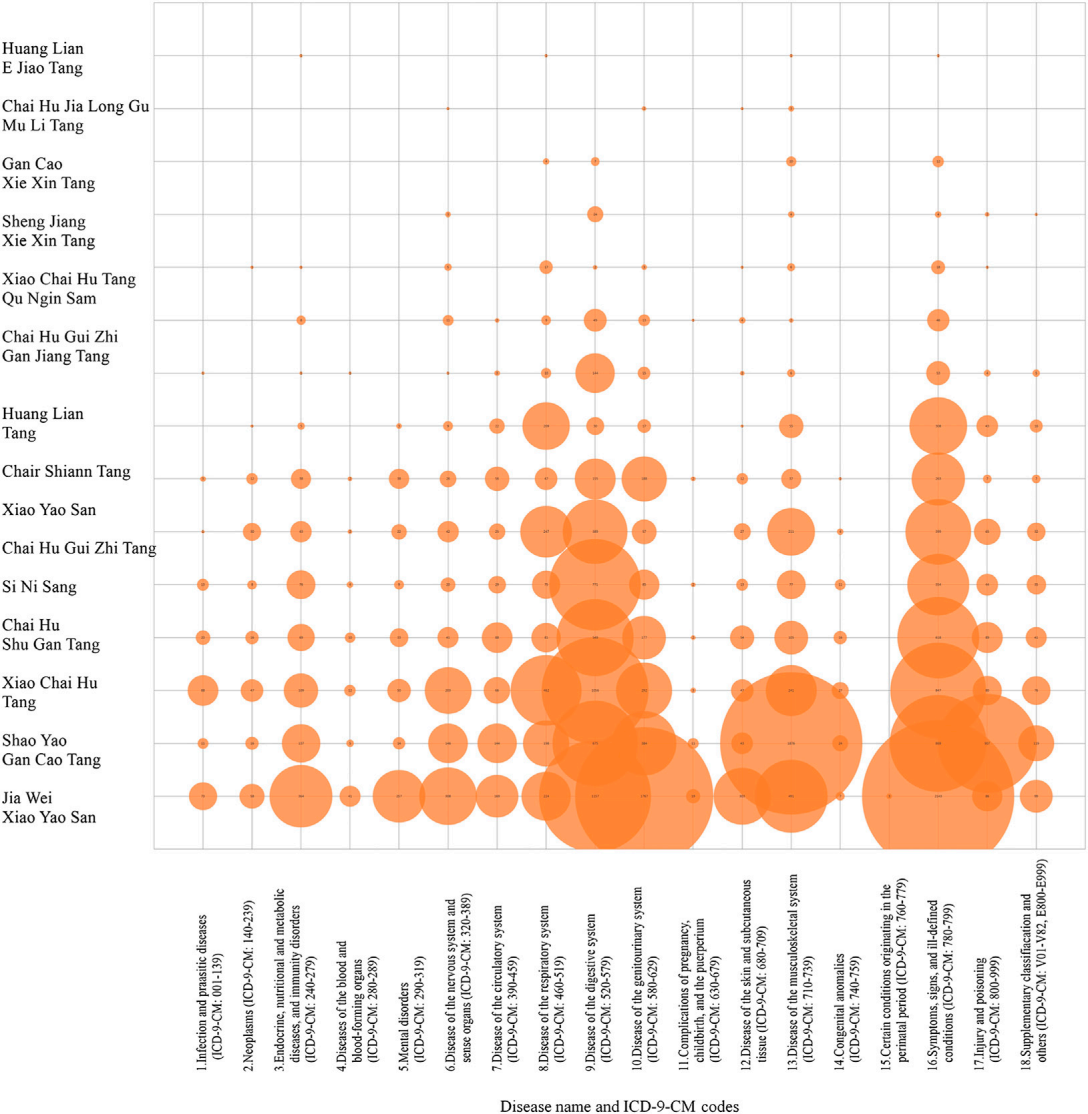
Prescription frequency of constituent herbs of harmonizing formulas categorized by systematic disease and related health problems in patients with chronic kidney disease.

As Figure 1 presents, the three most frequently prescribed herbs for harmonizing formulas during the observed period were Jia Wei Xiao Yao San (Composition: *Angelica sinensis* (Oliv.) Diels; *Atractylodes macrocephala* Koidz.; *Paeonia lactiflora* Pall.; *Bupleurum chinense* DC.; *Wolfiporia extensa* (Peck) Ginns (syn. *Poria cocos* (Schwein.) F. A. Wolf); *Glycyrrhiza uralensis* Fisch ex DC.; *Paeonia x suffruticosa* Andrews; *Gardenia jasminoides* J. Ellis; *Zingiber officinale* Roscoe; *Mentha canadensis* L. (syn. Mentha haplocalyx Briq.), Shao Yao Gan Cao Tang (Composition: *Paeonia lactiflora* Pall; *Glycyrrhiza uralensis* Fisch ex DC.), and Xiao Chai Hu Tang (Composition: *Bupleurum chinense* DC.; *Scutellaria baicalensis* Georgi, *Panax ginseng* C. A. Mey.; *Glycyrrhiza uralensis* Fisch ex DC.; *Pinellia ternata* (Thunb.) Makino; *Zingiber officinale* Roscoe; *Ziziphus jujuba* Mill.) (Additives and Feed, 2015; Selyutina and Polyakov, 2009). Frequent disease categories for Jia Wei Xiao Yao San prescriptions were symptoms, signs, and ill-defined conditions (*n* = 2,143 times); genitourinary disease (*n* = 1,767 times); and diseases of the digestive system (*n* = 1,157 times). The most frequent disease category for Shao Yao Gan Cao Tang prescriptions was diseases of the musculoskeletal system (*n* = 1,876 times). Furthermore, the most frequent disease category for Xiao Chai Hu Tang prescription was diseases of the digestive system (*n* = 1,056 times).

### Factors Associated With Harmonizing Formulas Prescription

Table 3 presents the associations of baseline patient characteristics with harmonizing formula prescriptions. Patients who were young (adjusted OR: 0.98, 95% CI: 0.97–0.98); female (1.79, 1.68–1.91); had high insurance amounts (range of adjusted OR: 1.14–1.31); lived in central Taiwan (1.26, 1.16–1.37), southern Taiwan (1.20, 1.11–1.30), or urban areas (1.17, 1.07–1.26); did not have diabetes (1.20, 1.06–1.36), hypertension (1.38, 1.27–1.50), or cerebrovascular disease (1.34, 1.14–1.56); had lower disease severity (0.85, 0.83–0.88); used NSAIDs (1.65, 1.54–1.78); and used analgesic drugs other than NSAIDs (1.47, 1.35–1.59) were more likely to have harmonizing formula prescriptions.

**TABLE 3:** Factors associated with harmonizing formula prescription in patients with chronic kidney disease.

	Adjusted odds ratio (95%CI)	*p* Value
Age, y	0.98 (0.97–0.98)	<0.001
Female (male as References)	1.79 (1.68–1.91)	<0.001
Insurance amount, NT$		
Fixed premium or dependent	1.00 [reference]	
<20,000	1.14 (1.04–1.24)	0.004
20,000–39,999	1.25 (1.12–1.40)	<0.001
≧39,999	1.31 (1.15–1.50)	<0.001
Region		
North	1.00 [reference]	
Center	1.26 (1.16–1.37)	<0.001
South	1.20 (1.11–1.30)	<0.001
East	0.88 (0.73–1.07)	0.20
Urbanization		
Rural	1.00 [reference]	
Urban	1.17 (1.07–1.26)	<0.001
Comorbidities (yes as References)		
No diabetes	1.20 (1.06–1.36)	0.004
No hypertension	1.38 (1.27–1.50)	<0.001
No COPD	0.87 (0.75–1.01)	0.06
No cerebrovascular disease	1.34 (1.14–1.56)	<0.001
Charlson score, point	0.85 (0.83–0.88)	<0.001
Confounding drugs (none as References)		
Diabetic drugs	1.08 (0.97–1.20)	0.17
Antihypertensive drugs	1.08 (0.99–1.17)	0.07
NSAIDs	1.65 (1.54–1.78)	<0.001
Analgesic drugs other than NSAIDs	1.47 (1.35–1.59)	<0.001

Abbreviations: COPD, chronic obstructive pulmonary disease; CI, confidence interval; NSAIDs, nonsteroidal anti-inflammatory drugs; NT$, New Taiwan dollar.

Multiple logistic regression analysis starts with a model that contains all variables in Table 1 and backward eliminates insignificant variables by a significance level of 0.2. NT$30 equals approximately US$1.

## Discussion

The current study demonstrated that one-fifth of patients with CKD have been prescribed harmonizing formulas, which were associated with risk reduction of ESRD in our previous study (Lin et al., 2015). Symptoms, signs, and ill-defined conditions, diseases of the digestive system, and musculoskeletal system diseases were the three most frequent disease classifications for prescribing harmonizing formulas. Patients with CKD who were young, female, had high premiums, lived in central or southern Taiwan or urban areas, did not have comorbidities (acute coronary syndrome, diabetes, hypertension, or cerebrovascular disease), had lower disease severity, used NSAIDs, and analgesic drugs other than NSAIDs were more likely to have harmonizing formulas prescriptions.

Nearly half of incident CKD patients used conventional CHM. Harmonizing formulas accounted for 46.1% of prescriptions. Although the efficacy of harmonizing formulas in reducing depression and improving survival in patients with liver cancers and systemic lupus erythematosus have been reported in studies (Liao et al., 2015; Ma et al., 2016), few studies have paid attention to prescription patterns and outcomes in treating patients with CKD (Yang et al., 2014; Chen et al., 2018). Combined with the results of Yang et al. (2014), Chen et al. (2018), and our previous findings, the current study indicated that one harmonizing formula, Jia Wei Xiao Yao San, is the main prescription for patients with late CKD. This formula potentially improves patient renal function after short- or long-term observation (Yang et al., 2014; Chen et al., 2015; Lin et al., 2015). Jia Wei Xiao Yao San is traditionally used to relieve stagnation in liver qi, reduce depression symptoms, and improve spleen qi deficiency. Although the mechanisms of delaying ESRD are complex and worthy of further study, Jia Wei Xiao Yao San may affect renal clinical outcome by improving depressive symptoms, which are a novel predictor of accelerated eGFR decrease, dialysis therapy initiation, death, or hospitalization (Chen et al., 2015; Shirazian et al., 2017). We are unsure of the effect harmonizing formulas have in relieving uremic symptoms or slowing renal progression.

Saikosaponins are the main bioactive compounds in the dry root of *Bupleurum chinense* DC., which is the chief herb of harmonizing formulas. The recent comprehensive review shows that it is promising to improve renal cell injuries by mediating either activity of anti-oxidant-dant enzymes (superoxide dismutase, catalase, glutathione peroxidase) and heat shock protein 72, SirT3, or mitogen-activated protein kinase and nuclear factor-κB (Li et al., 2018b). Other animal studies further proved the roles of saikosaponins on diabetes nephropathy, glomerulonephritis, and renal fibrosis (Li et al., 2005; Lin et al., 2012; Ren et al., 2020). In addition, other chief herb likes *Paeonia lactiflora* Pall. within Shao Yao Gan Cao Tang provides possibilities on kidney benefits through improving hemorheological abnormalities and protecting vascular endothelial function (Li et al., 2020; Tan et al., 2020). Although these active compounds of saikosaponins bring insights for kidney injuries, a previous animal study proposes their safety concerns about liver injury when treating mice with saikosaponins higher than13 g/kg (Yao et al., 2013; Li X. et al., 2018). Anyway, more studies ensuring the efficacy and safety of harmonizing formulas for humans are needed.

We attempted to use the ICD-9-CM as a reference for identifying the indications of harmonizing formula prescription in CKD patients. CHM has its own historical and systematic philosophy of symptom differentiation (Bian Zheng) to assess, explicate, diagnose, and treat patient symptoms. In Werstern medicine, numerous symptoms are either unrecognized or suboptimally managed by clinical care workers in CKD patients not requiring dialysis (van der Veer et al., 2017). Brown et al. assessed 283 patients with stage 1–5 CKD and reported that tiredness (81%; 95% CI: 76.0–85.6), sleep disturbance (70%; 64.3–75.3), and pain in bones or joints (69%; 63.4–74.6) were the most common symptoms regardless of CKD stage (Brown et al., 2017). Loss of appetite, nausea, vomiting, fatigue, and edema are common symptoms in late-stage CKD, which are similar to “spleen deficiency syndrome” in CHM and tend to involve the digestive system. In addition, local pain, weakness in the loin and knee, and calf cramps are frequently encountered CKD symptoms, which are similar to “liver–kidney insufficiency syndromes” in CHM and tend to involve the musculoskeletal system. Jia-Wei-Xiao-Yao-San has been demonstrated that it can adjust the abnormal gastric motility and gastric myoelectrical activity of patients with functional dyspepsia (Qu et al., 2010). Shao Yao Gan Cao Tang, a frequently prescribed harmonizing formula in CKD, is efficacy in relieving muscle pain or skeletal muscle tremors in patients undergoing hemodialysis or with cirrhosis or lumbar spinal stenosis (Hinoshita et al., 2003; Ota et al., 2020). Our findings suggest symptoms involving the digestive system composite the main syndromes for typical harmonizing formulas prescription (Table 2). These main syndromes have been listed in our Supplementary Table S1 with their corresponding formulas. Radix Bupleuri (*Bupleurum chinense* DC.) is the main chief herb within the listed composition of many harmonizing formulas identified in our study (Supplementary Table S2) (Xie et al., 2013; Ji et al., 2014; Tian et al., 2014; Zhang et al., 2017; Pastorino et al., 2018; Stanisiere et al., 2018; Mao et al., 2019; Wang et al., 2019; Yang et. al, 2020). Other candidates of chief herbs within each harmonizing formula, and their pharmacological actions and toxicity assessment are also listed in Supplementary Tables S2–S4. In brief, the harmonizing formulas probably diminish the symptoms of CKD patients through improving both digestive and musculoskeletal systems.

Female sex, low prevalence of comorbidity, and high use of analgesic drugs were associated with higher prescription frequency for harmonizing formulas in CKD. Although the causal relationships are difficult to establish in this study, a possible explanation is that prevalence of pain for females is high, and they are more likely to be aware of pain and receive relevant treatments (Fillingim, 2000; Johannes et al., 2010). NSAIDs are commonly used for pain control in clinical practice, but caution should be exercised when they are applied in CKD because they can induce more severe renal injuries (Luciano et al., 2015). How harmonizing formulas interact with NSAIDs for pain control in patients with CKD remains unclear. Thus, more research is required on this combination therapy to study its efficacy in pain control in CKD and preventing further renal injury.

Our study has some advantages. First, assessments of Chinese herbal formulas in the study were drawn from a nationwide health insurance database with highly comprehensive records of CHM prescriptions. Second, CHM in this study was prescribed by quality assurance physicians who were educated in the same system and accredited by Taiwan’s government; this strengthens the reliability of symptomatic differentiation and accuracy of disease diagnostic coding. Third, Taiwan’s NHI is one of the few national insurance programs that reimburse both Western medicine and CHM, providing an opportunity to explore the foundational philosophies of these two different modes for treating certain diseases. However, some limitations must be declared. First, the lack of laboratory and patient-reported data in the claims database prevents us from exploring the possible mechanisms of harmonizing formulas on health outcomes such as emotion, pain, and renal function. In addition, our study lacks original indications for these harmonizing formulas prescription from CHMs. Using the ICD-9-CM diagnosis system to identify disease classification of CHM prescriptions may not appropriately reflect the indications of CHM formulas. Third, CKD usually accompanies by different chronic conditions. The indications of harmonizing formulas prescription identified in our study may not be direct from CKD, and one should interpret the result with caution. Finally, our results were from the Asian population covered by the NHI program in Taiwan and derived from older sample data, limiting their generalizability.

## Conclusion

In conclusion, this study determined that harmonizing formulas are commonly used in treating CKD for symptoms, signs, and ill-defined conditions; genitourinary diseases; and digestive system diseases. Patients who were young, female, had fewer comorbidities, and used analgesic drugs were more likely to be prescribed harmonizing formulas, which suggests that more research on the efficacy of Western medicine and CHM in patients with CKD and these characteristics is required.

## Data Availability

The datasets presented in this article are not readily available because these Data used in our study should be acquired through formal application to the Health and Welfare Data Science Center, Department of Statistics, Ministry of Health and Welfare, Taiwan. Requests to access the datasets should be directed to https://dep.mohw.gov.tw/DOS/np-2497-113.html.

## References

[B1] Additives E. P. O. Feed P. O. S. U. I. A. (2015). Scientific Opinion on the safety and efficacy of glycyrrhizic acid ammoniated (chemical group 30, miscellaneous substances) when used as a flavouring for all animal species. EFSA J. 13, 3971. 10.2903/j.efsa.2015.3971

[B2] Brown S. A. Tyrer F. C. Clarke A. L. Lloyd-Davies L. H. Stein A. G. Tarrant C. (2017). Symptom burden in patients with chronic kidney disease not requiring renal replacement therapy. Clin. Kidney J. 10, 788–796. 10.1093/ckj/sfx057 29225808 PMC5716066

[B3] Cao M. (2000). [Wang Ang and his Variorum of medical recipes (Yi fang ji jie)]. Zhonghua Yi Shi Za Zhi 30, 179–181. 11624695

[B4] Chan K. (1995). Progress in traditional Chinese medicine. Trends Pharmacol. Sci. 16, 182–187. 10.1016/s0165-6147(00)89019-7 7652926

[B5] Chen J. K. Chen T. T. (2009). Chinese herbal formulas and applications. City of Industry, CA, Art of Medicine Press.

[B6] Chen W. Chen H.-Y. Yang Y.-H. Yang S.-H. Yang C.-W. Wu Y.-H. (2018). An investigation of the prescription patterns of Chinese herbal products for chronic glomerulonephritis patients: a hospital-based cross-sectional study, Evidence-Based Complement. Altern. Med. 1-11. 10.1155/2018/5080764 PMC6276402 30581484

[B7] Chen Y.-L. Lee C.-Y. Huang K.-H. Kuan Y.-H. Chen M. (2015). Prescription patterns of Chinese herbal products for patients with sleep disorder and major depressive disorder in Taiwan. J. Ethnopharmacology 171, 307–316. 10.1016/j.jep.2015.05.045 26068429

[B8] Collins A. J. Chen S.-C. Gilbertson D. T. Foley R. N. (2009). CKD surveillance using administrative data: impact on the health care system. Am. J. Kidney Dis. 53, S27–S36. 10.1053/j.ajkd.2008.07.055 19231758

[B9] Fillingim R. B. (2000). Sex, gender, and pain: women and men really are different. Curr. Rev. Pain 4, 24–30. 10.1007/s11916-000-0006-6 10998712

[B10] Hinoshita F. Ogura Y. Suzuki Y. Hara S. Yamada A. Tanaka N. (2003). Effect of orally administered shao-yao-gan-cao-tang (Shakuyaku-kanzo-to) on muscle cramps in maintenance hemodialysis patients: a preliminary study. Am. J. Chin. Med. 31, 445–453. 10.1142/s0192415x03001144 12943175

[B11] Hsieh C.-F. Chang H.-C. Huang S.-L. Chen C.-L. Chen W.-T. Yang C.-C. (2017). Prescribed renoprotective Chinese herbal medicines were associated with a lower risk of all-cause and disease-specific mortality among patients with chronic kidney disease: a population-based follow-up study in taiwan, Evidence-Based Complement. Altern. Med. 2017, 5632195. 10.1155/2017/5632195 PMC5535732 28798802

[B12] Hsieh C. F. Huang S. L. Chen C. L. Chen W. T. Chang H. C. Yang C. C. (2014). Non-aristolochic acid prescribed Chinese herbal medicines and the risk of mortality in patients with chronic kidney disease: results from a population-based follow-up study. BMJ open 4, e004033. 10.1136/bmjopen-2013-004033 PMC3931999 24561496

[B13] Hsu P.-C. Tsai Y.-T. Lai J.-N. Wu C.-T. Lin S.-K. Huang C.-Y. (2014). Integrating traditional Chinese medicine healthcare into diabetes care by reducing the risk of developing kidney failure among type 2 diabetic patients: a population-based case control study. J. Ethnopharmacology 156, 358–364. 10.1016/j.jep.2014.08.029 25178949

[B14] Jha V. Garcia-Garcia G. Iseki K. Li Z. Naicker S. Plattner B. (2013). Chronic kidney disease: global dimension and perspectives. The Lancet 382, 260–272. 10.1016/s0140-6736(13)60687-x 23727169

[B15] Ji X. Huang B. Wang G. Zhang C. (2014). The ethnobotanical, phytochemical and pharmacological profile of the genus Pinellia. Fitoterapia 93, 1–17. 10.1016/j.fitote.2013.12.010 24370664

[B16] Jiang M. Lu C. Zhang C. Yang J. Tan Y. Lu A. (2012). Syndrome differentiation in modern research of traditional Chinese medicine. J. Ethnopharmacology 140, 634–642. 10.1016/j.jep.2012.01.033 22322251

[B17] Johannes C. B. Le T. K. Zhou X. Johnston J. A. Dworkin R. H. (2010). The prevalence of chronic pain in United States adults: results of an Internet-based survey. The J. Pain 11, 1230–1239. 10.1016/j.jpain.2010.07.002 20797916

[B18] Levey A. S. Coresh J. Bolton K. Culleton B. Harvey K. S. Ikizler T. A. (2002). K/DOQI clinical practice guidelines for chronic kidney disease: evaluation, classification, and stratification. Am. J. Kidney Dis. 39, S1-266. 11904577

[B19] Levin A. Stevens P. E. Bilous R. W. Coresh J. De Francisco A. L. De Jong P. E. (2013). Kidney Disease: improving Global Outcomes (KDIGO) CKD Work Group. KDIGO 2012 clinical practice guideline for the evaluation and management of chronic kidney disease. Kidney Int. Supplements 3, 1–150. 10.1038/kisup.2012.73 23732715

[B20] Li P. Gong Y. Zu N. Li Y. Wang B. Shimizu F. (2005). Therapeutic mechanism of Saikosaponin-d in anti-Thy1 mAb 1-22-3-induced rat model of glomerulonephritis. Nephron Exp. Nephrol. 101, e111–e118. 10.1159/000087437 16103731

[B21] Li P. Shen J. Wang Z. Liu S. Liu Q. Li Y. (2020). Genus Paeonia: a comprehensive review on traditional uses, phytochemistry, pharmacological activities, clinical application, and toxicology. J. Ethnopharmacology 269, 113708. 10.1016/j.jep.2020.113708 33346027

[B22] Li X.-Q. Song Y.-N. Wang S.-J. Rahman K. Zhu J.-Y. Zhang H. (2018b). Saikosaponins: a review of pharmacological effects. J. Asian Nat. Prod. Res. 20, 399–411. 10.1080/10286020.2018 29726699

[B23] Li X. Li X. Huang N. Liu R. Sun R. (2018a). A comprehensive review and perspectives on pharmacology and toxicology of saikosaponins. Phytomedicine 50, 73–87. 10.1016/j.phymed.2018.09.174 30466994 PMC7126585

[B24] Liao Y.-H. Lin C.-C. Lai H.-C. Chiang J.-H. Lin J.-G. Li T.-C. (2015). Adjunctive traditional Chinese medicine therapy improves survival of liver cancer patients. Liver Int. 35, 2595–2602. 10.1111/liv.12847 25875878

[B25] Lin C. C. Lin L. T. Yen M. H. Cheng J. T. Hsing C. H. Yeh C. H. (2012). Renal protective effect of Xiao-Chai-Hu-Tang on diabetic nephropathy of type 1-diabetic mice. Evid. Based Complement. Alternat Med. 2012, 984024. 10.1155/2012/984024 22474533 PMC3310293

[B26] Lin H.-L. Lin M.-Y. Tasi C.-H. Wang Y.-H. Chen C.-J. Hwang S.-J. C. (2020). Harmonizing formula prescription patterns for chronic kidney disease: a population-based cross-sectional study. [Preprint], 10.21203/rs.3.rs-20650/v1 PMC8117089 33995002

[B27] Lin M.-Y. Chiu Y.-W. Chang J.-S. Lin H.-L. Lee C. T.-C. Chiu G.-F. (2015). Association of prescribed Chinese herbal medicine use with risk of end-stage renal disease in patients with chronic kidney disease. Kidney Int. 88, 1365–1373. 10.1038/ki.2015.226 26244923

[B28] Lu Z. Zeng Y. Lu F. Liu X. Zou C. (2015). Rhubarb enema attenuates renal tubulointerstitial fibrosis in 5/6 nephrectomized rats by alleviating indoxyl sulfate overload. PloS one 10, e0144726. 10.1371/journal.pone.0144726 26671452 PMC4684395

[B29] Luciano R. Perazella M. A. UpToDate (2015). in NSAIDs: acute kidney injury (acute renal failure) (Waltham, MA: UpToDate, BasowDS).

[B30] Ma Y. C. Lin C. C. Li C. I. Chiang J. H. Li T. C. Lin J. G. (2016). Traditional Chinese medicine therapy improves the survival of systemic lupus erythematosus patients, Semin. Arthritis Rheum., 45(5). 596–603. 10.1016/j.semarthrit.2015.09.006 26522135

[B31] Mao Q.-Q. Xu X.-Y. Cao S.-Y. Gan R.-Y. Corke H. Li H.-B. (2019). Bioactive compounds and bioactivities of ginger (Zingiber officinale Roscoe). Foods 8(6), 185. 10.3390/foods8060185 PMC6616534 31151279

[B32] Ota K. Fukui K. Nakamura E. Oka M. Ota K. Sakaue M. (2020). Effect of Shakuyaku‐kanzo‐to in patients with muscle cramps: a systematic literature review. J. Gen. Fam. Med. 21, 56–62. 10.1002/jgf2.302 32489757 PMC7260166

[B33] Pastorino G. Cornara L. Soares S. Rodrigues F. Oliveira M. B. P. (2018). Liquorice (*Glycyrrhiza* glabra): a phytochemical and pharmacological review. Phytotherapy Res. 32, 2323–2339. 10.1002/ptr.6178 PMC7167772 30117204

[B34] Qu Y. Gan H. Q. Mei Q. B. Liu L. (2010). Study on the effect of Jia-Wei-Xiao-Yao-San decoction on patients with functional dyspepsia. Phytother Res. 24, 245–248. 10.1002/ptr.2920 19610028

[B35] Ren D. Luo J. Li Y. Zhang J. Yang J. Liu J. (2020). Saikosaponin B2 attenuates kidney fibrosis via inhibiting the Hedgehog Pathway. Phytomedicine 67, 153163. 10.1016/j.phymed.2019.153163 31901891

[B36] Selyutina O. Y. Polyakov N. (2019). Glycyrrhizic acid as a multifunctional drug carrier–From physicochemical properties to biomedical applications: a modern insight on the ancient drug. Int. J. Pharmaceutics 559, 271–279. 10.1016/j.ijpharm.2019.01.047 PMC7126914 30690130

[B37] Shirazian S. Grant C. D. Aina O. Mattana J. Khorassani F. Ricardo A. C. (2017). Depression in chronic kidney disease and end-stage renal disease: similarities and differences in diagnosis, epidemiology, and management. Kidney Int. Rep. 2, 94–107. 10.1016/j.ekir.2016.09.005 29318209 PMC5720531

[B38] Stanisiere J. Mousset P.-Y. Lafay S. (2018). How safe is ginger rhizome for decreasing nausea and vomiting in women during early pregnancy? Foods 7, 50. 10.3390/foods7040050 PMC5920415 29614764

[B39] Su X. Yao Z. Li S. Sun H. (2016). Synergism of Chinese herbal medicine: illustrated by danshen compound, Evid Based. Complement. Altern. Med. 2016, 7279361. 10.1155/2016/7279361 PMC4846759 27190537

[B40] Tan Y.-Q. Chen H.-W. Li J. Wu Q.-J. (2020). Efficacy, chemical constituents, and pharmacological actions of Radix Paeoniae rubra and Radix Paeoniae alba. Front. Pharmacol. 11, 1054. 10.3389/fphar.2020.01054 32754038 PMC7365904

[B41] Tian T. Chen H. Zhao Y.-Y. (2014). Traditional uses, phytochemistry, pharmacology, toxicology and quality control of Alisma orientale (Sam.) Juzep: a review. J. Ethnopharmacology 158, 373–387. 10.1016/j.jep.2014.10.061 25446590

[B42] Van Der Veer S. N. Aresi G. Gair R. (2017). Incorporating patient-reported symptom assessments into routine care for people with chronic kidney disease. Clin. Kidney J. 10, 783–787. 10.1093/ckj/sfx106 29250324 PMC5721341

[B43] Wang J. Wang L. Lou G.-H. Zeng H.-R. Hu J. Huang Q.-W. (2019). Coptidis Rhizoma: a comprehensive review of its traditional uses, botany, phytochemistry, pharmacology and toxicology. Pharm. Biol. 57, 193–225. 10.1080/13880209.2019.1577466 30963783 PMC6461078

[B44] Xie X. Chang X. Chen L. Huang K. Huang J. Wang S. (2013). Berberine ameliorates experimental diabetes-induced renal inflammation and fibronectin by inhibiting the activation of RhoA/ROCK signaling. Mol. Cell Endocrinol. 381, 56–65. 10.1016/j.mce.2013.07.019 23896433

[B45] Yang T.-H. Chen H. Yang S.-H. Lin Y.-H. Fang J.-T. Hung C.-C. (2014). Utilization pattern for traditional Chinese medicine among late stage chronic kidney disease patients: a hospital-based cross-sectional study. J. Chin. Med. 25, 41–58. 10.1155/2018/1706517

[B46] Yang X.-Z. Wei W. (2020). CP-25, a compound derived from paeoniflorin: research advance on its pharmacological actions and mechanisms in the treatment of inflammation and immune diseases. Acta Pharmacologica Sinica 41, 1387–1394. 10.1038/s41401-020-00510-6 32884075 PMC7656585

[B47] Yao R.-Y. Zou Y.-F. Chen X.-F. (2013). Traditional use, pharmacology, toxicology, and quality control of species in genus Bupleurum L. Chin. Herbal Medicines 5, 245–255. 10.1016/s1674-6384(13)60036-2 PMC7129159 32288759

[B48] Yeung W. F. Chung K. F. Ng K. Y. Yu Y. M. Zhang S. P. Ng B. F. (2015). Prescription of Chinese herbal medicine in pattern-based traditional Chinese medicine treatment for depression: a systematic review. Evid. Based Complement. Alternat Med. 2015, 160189. 10.1155/2015/160189 26180532 PMC4477207

[B49] Zeng L.-F. Cao Y. Wang L. Dai Y.-K. Hu L. Wang Q. (2017). Role of medicinal plants for liver-qi regulation adjuvant therapy in post-stroke depression: a systematic review of literature. Phytother. Res. 31, 40–52. 10.1002/ptr.5740 27762458

[B50] Zhang L. L. Xu W. Xu Y. L. Chen X. Huang M. Lu J. J. (2017). Therapeutic potential of Rhizoma Alismatis: a review on ethnomedicinal application, phytochemistry, pharmacology, and toxicology. Ann. New York Acad. Sci. 1401, 90–101. 10.1111/nyas.13381 28662316

[B51] Zhao T. Sun S. Zhang H. Huang X. Yan M. Dong X. (2016). Therapeutic effects of tangshen formula on diabetic nephropathy in rats. PloS one 11, e0147693. 10.1371/journal.pone.0147693 26807792 PMC4726711

[B52] Zhong J. Yang H.-C. Fogo A. B. (2017). A perspective on chronic kidney disease progression. Am. J. Physiology-Renal Physiol. 312, F375–F384. 10.1152/ajprenal.00266.2016 PMC5374308 27974318

