# Supplementary material for: Harmonizing Formula Prescription Patterns in Patients With Chronic Kidney Disease: A Population-Based Cross-Sectional Study
Source: Front Pharmacol. 2021 Apr 29;12:573145. doi: 10.3389/fphar.2021.573145 (PMC8117089; doi:10.3389/fphar.2021.573145)
Supplement: Supplementary file 1 [file datasheet1.docx]

Table S1. Main Chinese herb medicine (CHM) syndrome for harmonizing formulas and corresponding common symptoms

| CHM syndrome^*^ | Symptom | Typical harmonizing formulas |
| --- | --- | --- |
| Shaoyang^#^ | Constipation, nausea, vomiting, decreased appetite, muscle cramps, lightheadedness or dizziness, feeling tired or lack of energy, dry mouth, chest pain, headache, difficulty concentrating. | Xiao Chai Hu Tang, Chai Hu Gui Zhi Tang, Chair Shiann Tang, Chai Hu Gui Zhi Gan Jiang Tang, Xiao Chai Hu Tang Qu Ngin Sam, Chai Hu Jia Long Gu Mu Li Tang |
| Liver and Spleen disharmonies^#,^^ | Nausea, vomiting, diarrhea, decreased appetite, shortness of breath, muscle soreness, muscle pain, skeletal muscle tremors, difficulty concentrating, worrying, feeling nervous, trouble falling asleep, trouble staying asleep, feeling irritable, feeling sad, depression, feeling anxious, decreased interest in sex, difficulty becoming sexually aroused. | Jia Wei Xiao Yao San, Xiao Yao San, Si Ni Sang, Chai Hu Shu Gan Tang |
| Intestine and Stomach disharmonies^#^ | Nausea, vomiting, diarrhea, decreased appetite, difficulty concentrating, nocturia. | Sheng Jiang Xie Xin Tang, Gan Cao Xie Xin Tang, Huang Lian Tang, Huang Lian E Jiao Tang, Shao Yao Gan Cao Tang |

^*^ There are more than 728 syndroms commonly used in CHM.

^#^ belongs to the main category of sleen difficiency syndrome.

^^^contains to the category of liver–kidney insufficiency syndrome.

Table S2. Composition and principle herb of harmonizing formulas

| Chinese name  (No. of prescription) | Composition | Chief/principal herb |
| --- | --- | --- |
| Jia Wei Xiao Yao San  (n=7, 569 times) | *Angelica sinensis (*Oliv.*)* Dielss, 4 g; *Atractylodes macrocephala* Koidz., 4 g; *Paeonia lactiflora* Pall., 4 g; *Bupleurum chinense* DC., 4 g; *Poria cocos (*Schwein.*)* F.A.Wolf, 4 g; *Glycyrrhiza uralensis* Fisch, 2 g; *Paeonia suffruticosa* Andrewss, 2.5 g; *Gardenia jasminoides* J.Ellis, 2.5 g; *Zingiber officinale* Roscoe, 4 g; *Mentha haplocalyx* Briq, 2 g. | *Bupleurum chinense* DC. |
| Shao Yao Gan Cao Tang  (n=5,579 times) | *Paeonia lactiflora* Pall., 12 g; *Glycyrrhiza uralensis* Fisch, 12 g; | *Paeonia lactiflora* Pall. |
| Xiao Chai Hu Tang  (n=3,761 times) | *Bupleurum chinense* DC., 8 g; *Scutellaria baicalensis* Georgi, 3 g; *Panax ginseng* C. A. Mey., 3 g; *Glycyrrhiza uralensis* Fisch, 3 g; *Pinellia ternata (*Thunb.*)* Breitenb., 5 g; *Zingiber officinale* Roscoe, 3 g; *Ziziphus jujuba* Mill., 2 g. | *Bupleurum chinense* DC. |
| Chai Hu Shu Gan Tang  (n=2,009 times) | *Bupleurum chinense* DC., 3.2 g; *Citrus reticulata* Blanco, 3.2 g; *Ligusticum chuanxiong* Hort., 2.4 g; *Paeonia lactiflora* Pall., 2.4 g; *Citrus aurantium* L., 2.4 g; *Cyperus rotundus* L., 2.4 g; *Glycyrrhiza uralensis* Fisch, 0.8 g. | *Bupleurum chinense* DC. |
| Si Ni Sang  (n=1,626 times) | *Glycyrrhiza uralensis* Fisch, 6 g; *Citrus sinensis* Osbeck, 6 g; *Bupleurum chinense* DC., 6 g; *Paeonia lactiflora* Pall., 6 g. | *Bupleurum chinense* DC. |
| Chai Hu Gui Zhi Tang  (n=1,596 times) | *Cinnamomum cassia* (L.) J. Presl., 3 g; *Scutellaria baicalensis* Georgi, 3 g; *Panax ginseng* C. A. Mey., 3 g; *Glycyrrhiza uralensis* Fisch, 2 g; *Pinellia ternata (*Thunb.*)* Breitenb., 5 g; *Paeonia lactiflora* Pall., 3 g; *Ziziphus jujuba* Mill., 2 g; *Zingiber officinale* Roscoe, 3 g; *Bupleurum chinense* DC., 8 g. | *Bupleurum chinense* DC. |
| Xiao Yao San  (n=894 times) | *Glycyrrhiza uralensis* Fisch, 2 g; *Paeonia lactiflora* Pall., 4 g; *Bupleurum chinense* DC., 4 g; *Angelica sinensis (*Oliv.*)* Dielss, 4 g; *Atractylodes macrocephala* Koidz., 4 g; *Bupleurum chinense* DC., 4 g; *Zingiber officinale* Roscoe, 4 g; *Mentha haplocalyx* Briq, 2 g. | *Bupleurum chinense* DC. |
| Chair Shiann Tang  (n=719 times) | *Pinellia ternata (*Thunb*.)* Breitenb., 9 g; *Trichosanthes kirilowii* Maxim., 6 g; *Bupleurum chinense* DC., 6 g; *Coptis chinensis* Franch., 3 g; *Scutellaria baicalensis* Georgi, 3 g; *Panax ginseng* C. A. Mey., 3 g; *Glycyrrhiza uralensis* Fisch, 1.5 g; *Zingiber officinale* Roscoe, 3 g; *Ziziphus jujuba* Mill, 3 g. | *Pinellia ternata (Thunb.)* Breitenb |
| Huang Lian Tang  (n=246 times) | *Coptis chinensis* Franch., 4.5 g; *Glycyrrhiza uralensis* Fisch, 4.5 g; *Zingiber officinale* Roscoe, 4.5 g; *Cinnamomum cassia* (L.) J. Presl., 4.5 g; *Panax ginseng* C. A. Mey., 3 g; *Pinellia ternata* (Thunb.) Breitenb., 6 g; *Ziziphus jujuba* Mill, 3 g. | *Coptis chinensis* Franch.; *Zingiber officinale* Roscoe |
| Chai Hu Gui Zhi Gan Jiang Tang  (n=145 times) | *Bupleurum chinense* DC., 24 g; *Trichosanthes kirilowii* Maxim., 12 g; *Cinnamomum cassia* (L.) J. Presl., 9 g; *Scutellaria baicalensis* Georgi, 9 g; *shell of Crassostrea gigas (*Thunberg*) or Crassostrea rivularis (*Gould*)*, 6 g; *Zingiber officinale* Roscoe, 6 g; *Glycyrrhiza uralensis* Fisch, 6 g. | *Bupleurum chinense* DC. |
| Xiao Chai Hu Tang Qu Ngin Sam  (n=55 times) | *Bupleurum chinense* DC., 3.5 g; *Scutellaria baicalensis* Georgi, 1.5 g; *Panax ginseng* C. A. Mey, 1.5 g; *Pinellia ternata* (Thunb.) Breitenb., 2.5 g; *Glycyrrhiza uralensis* Fisch*,* 1.5 g; *Poria cocos* (Schwein.) F.A.Wolf, 2.2 g; *Polyporus umbellatus*, 2.2 g; *Alisma plantago-aquatica* subsp*. orientale* (Sam.) Sam., 3.0 g; *Atractylodes macrocephala* Koidz., 2.2 g; *Cinnamomum cassia* (L.) J. Presl., 1.5 g; *Zingiber officinale* Roscoe, 1.5 g; *Ziziphus jujuba* Mill, 1.5 g. | *Bupleurum chinense* DC.; *Alisma plantago-aquatica* subsp*. orientale* (Sam.) Sam. |
| Sheng Jiang Xie Xin Tang  (n=38 times) | *Zingiber officinale* Roscoe, 12 g; *Glycyrrhiza uralensis* Fisch, 9 g; *Panax ginseng* C. A. Mey, 9 g; *Zingiber officinale* Roscoe, 3 g; *Scutellaria baicalensis* Georgi, 9 g; *Pinellia ternata* (Thunb.) Breitenb., 12 g; *Coptis chinensis* Franch., 3 g; *Ziziphus jujuba* Mill 4 g. | *Zingiber officinale* Roscoe |
| Gan Cao Xie Xin Tang  (n=33 times) | *Glycyrrhiza uralensis* Fisch, 12 g; *Scutellaria baicalensis* Georgi, 9 g; *Zingiber officinale* Roscoe, 9 g; *Pinellia ternata* (Thunb.) Breitenb., 12 g; *Ziziphus jujuba* Mill, 12 g; *Coptis chinensis* Franch., 3 g; *Panax ginseng* C. A. Mey, 9 g. | *Glycyrrhiza uralensis* Fisch |
| Chai Hu Jia Long Gu Mu Li Tang (Chai Li Tang)  (n=7 times) | *Pinellia ternata* (Thunb.) Breitenb., 3 g; *Ziziphus jujuba* Mill, 2 g; *Bupleurum chinense* DC., 5 g; *Zingiber officinale* Roscoe, 2 g; *Panax ginseng* C. A. Mey, 2 g; *Ossis Mastodi Fossilia*, 2 g; *Cinnamomum cassia* (L.) J. Presl., 2 g; *Poria cocos (Schwein.)* F.A.Wolf, 2 g; *Rheum palmatum* L.*,* 2.5 g; *shell of Crassostrea gigas (*Thunberg*) or Crassostrea rivularis (*Gould*)*, 2 g; *Scutellaria baicalensis* Georgi, 2 g. | *Bupleurum chinense* DC. |
| Huang Lian E Jiao Tang  (n=4 times) | *Coptis chinensis* Franch., 2 g; *Scutellaria baicalensis* Georgi, 6 g; *Paeonia lactiflora* Pall., 6 g; *Egg yolk*, 2; *skin of Equus asinus* L*,* 9 g. | *Coptis chinensis* Franch*.* |

Table S3. Pharmacological actions of active compounds within harmonizing formulas

| Chief/principal herb within harmonizing formula | Active Compounds | Pharmacological actions |
| --- | --- | --- |
| *Bupleurum chinense* DC. | Saikosaponins (Li et al., 2018) | Anti-inflammatory activity, Immunomodulatory activity, Anti-liver cancer and the potential hepatotoxicity, Anticancer activity, Antidepressant activity, Antiepileptic activity, Neuropathic pain attenuation, Antiviral activity |
| *Paeonia lactiflora* Pall. | Paeoniflorin (Tan et al., 2020;Yang and Wei, 2020) | Anti-inflammatory action, Immunoregulatory effects, Antitumor activity, Protective effect on cardiac remodeling, Anti-inflammatory and immunoregulatory effects, Improvement of hemorheological abnormalities and protection on vascular endothelial function |
|  | Albiflorin (Tan et al., 2020) | Neuroprotective effect, Antidepressant effect, Sedative effect, Analgesic effect, Anticonvulsant effect |
| *Pinellia ternata* (Thunb.) Breitenb. (Ji et al., 2014) | Alkaloids and  Lectins | Anti-tumor activity |
|  | Alkaloids and proteins | Antiemetic activity |
|  | Pinellia lectins | Insecticidal activity |
|  | Organic acid | Antitussive activity |
|  | Pinelloside | Antimicrobial, antifungal and antiviral activities |
|  | *Pinellia* alkaloids | Sedative, hypnotic and anticonvulsive activities |
|  | Proteins of *P. ternata* | anti-early pregnancy effect: The inhibitory effect on the secretion of ovarian flavonoids and decreased levels of plasma progesterone may be responsible for miscarriage |
|  | *Pinellia* species | Anti-inflammatory, Analgesic, Anti-arrhythmic, Anti-hyperlipidemia activities, Blood circulation promotion, Reducing intraocular pressure and preventing the side effects of contrast agent |
| *Coptis chinensis* Franch. | *Berberine (BBR)；*  Other alkaloids(coptisine, palmatine, epiberberine, jatrorrhizine, magnoflorine,etc) | Inhibition of renal inflammation (Xie et al., 2013)  Anti-pathogenic microorganism activity: antiviral effect,  Antifungal effect (Wang et al., 2019).  Protective effects on the cardiovascular system: anti-atherosclerotic, anti-hyperlipidemic, anti-diabetic, Antihepatic steatototic effects, protective effect against ischaemic heart disease (Wang et al., 2019).  Antidiabetes: improving glucose metabolism, improving insulin resistance, improving pancreatic b cells and promoting the secretion of insulin, modulating gut microbiota (Wang et al., 2019).  Anticancer effect: inducing apoptosis, cell cycle arrest, inhibiting tumour metastasis (Wang et al., 2019). |
| *Zingiber officinale* Roscoe | Gingerol, and shogaol | Antioxidant activity, Anti-Inflammatory activity, Antimicrobial activity, Cytotoxicity, Neuroprotection, Cardiovascular protection, Antiobesity activity, antidiabetic activity, Antinausea and antiemetic activities, Protective Effects against respiratory disorders, hepatoprotective , antiallergic effects (Mao et al., 2019). |
| *Alisma orientale* (Sam.) Juzep | Alisol, and other extracts | Diuretic effect, Hypolipidemic activity, Hyperglycemic activity, Nephroprotective effect, Immuno-enhancing activity, Anti-inflammatory activity, Anti-tumor activity, Inhibiting the production of NO, Anti-complement activity, Anti-oxidative activity, Antiviral effect, Inhibition on angiotensin II, Antiplasmodial effect, Anti-hepatitis B virus activity, Antibacterial activity, Osteoclastogenesis inhibition, Hepatoprotective effect, Regulation the 5-HT3A receptors, Inhibition the adipocyte differentiation, Adrenergic mechanism, Hypouricemic effect (Tian et al., 2014). |
| *Glycyrrhiza uralensis* Fisch. | Glabridin | Neuroprotective activity, Sedative activity, Oestrogenic activity, Skin effects, Anticarcinogenic activity, Antimicrobial activity, Antioxidant activity, Anti‐inflammatory activity (Pastorino et al., 2018). |
|  | Glycyrrhizinic acid | Skin effects, Antiviral activity, Antimicrobial activity, Hepatoprotective activity, Anti‐inflammatory activity (Pastorino et al., 2018). |

Table S4. Toxicity assessment for using harmonizing formula

| Chief/principal herb within harmonizing formula | Sample source | Type of model | Specie | Dose and effect | Convert factor to human | Estimated human dose |
| --- | --- | --- | --- | --- | --- | --- |
| *Bupleurum chinense* DC. (Yao et al., 2013) | Total saponins | Acute | Rats | 2255.6 mg/(kg·d); LD_50_ | 6.25 | （2255.6÷6.25）× 60  ＝ 21653.8 mg/(60 kg.people body weight.d)  ＝ 21.7 g/(60 kg.people body weight.d) |
| *Paeonia lactiflora* Pall. (Li et al., 2020) | Aqueous extracts of the roots | Acute oral toxicity test | Rats | 2000 mg/(kg·Rat); No observed mortality | 6.25 | （2000÷6.25）× 60  ＝ 19200 mg/(60 kg.people body weight.d)  ＝ 19.2 g/(60 kg.people body weight.d) considered safe |
| *Coptis chinensis* Franch*.* (Wang et al., 2019) | Aqueous extracts of the rhizomes | Acute toxicity | Mice | 2.95 g/(kg·Mice); LD_50_ | 9.01 | （2.95÷9.01）× 60  ＝ 46.6 g/(60 kg.people body weight.d**)** |
|  | Aqueous extracts of the rhizomes | Sub-chronic toxicity | Rats | 1.88 g/kg·Rat; No-observed-adverse effect level | 6.25 | （1.88÷6.25）× 60  ＝ 18.0 g/(60 kg.people body weight.d) |
|  | Aqueous extracts of the rhizomes | Sub-chronic toxicity | Rats | 3.76 g/kg; No-observed-liver and lung damage | 6.25 | （3.76÷6.25）× 60  ＝ 36.1 g/(60 kg.people body weight.d) |
|  | Berberine | Acute toxicity | Mice | 713.57 mg/kg; LD_50_ | 9.01 | 4752 mg/(60 kg.people body weight.d) |
|  | Coptisine | Acute toxicity | Mice | 852.12 mg/kg; LD_50_ | 9.01 | 5674 mg/(60 kg.people body weight.d) |
|  | Palmatine | Acute toxicity | Mice | 1533.68 mg/kg; LD_50_ | 9.01 | 10213 mg/(60 kg.people body weight.d) |
|  | Epiberberine | Acute toxicity | Mice | 1360 mg/kg; LD_50_ | 9.01 | 9057 mg/(60 kg.people body weight.d) |
| *Alisma orientale* (Sam.) Juzep (Tian et al., 2014;Zhang et al., 2017) | The aqueous extract of RA | Sub-chronic toxicity | Rats | 33.3 g/kg Rat; Increased serum BUN and the renal enzyme γ -GT | 6.25 | (33.3÷6.25）× 60  ＝319.68 g/(60 kg.people body weight.d) |
|  | Triterpene-enriched extract of RA | Chronic toxicity | Rats | 1440 mg/kg/d; No observed mortality | 6.25 | (1.44÷6.25）× 60  ＝13.824 g/(60 kg.people body weight.d) |
| *Glycyrrhiza uralensis* Fisch.(Additives and Feed, 2015;Selyutina and Polyakov, 2019) | Glycyrrhizic acid | Acute toxicity | Rats | >610mg/kg; LD_50_ | 6.25 | （0.61÷6.25）× 60  > 5.856 g/(60 kg.people body weight.d) |
|  | European Food Safety Authority in 2015 concluded that glycyrrhizic acid ammoniated is safe at a concentration of 1 mg/kg complete feed for all species except chickens for fattening and laying hens ((Tian et al., 2014), 2015). | | | | | |
| *Pinellia ternata* (Thunb.) Breitenb. (Ji et al., 2014) | Suspension of raw *P. ternata* | Acute toxicity | Mice | 42.7 ± 1.27 g/kg; LD_50_ | 9.01 | （42.7± 1.27÷9.01）× 60  ＝ 275.89~292.8 g/(60 kg.people body weight.d) |
|  | The maximum dosage (MLD) values of water extract: 300.0 g/kg | - | - | - | - | This dose is equal to 2333.3 times of 70 kg people's daily dried medicinal herb expenses. |
| *Zingiber officinale* Roscoe (Stanisiere et al., 2018) | 6-shogaol | Acute/subacute toxicity | Mice | 687 mg/kg; Oral LD_50_ | 9.01 | （0.687÷9.01）× 60  ＝ 4.575 g/(60 kg.people body weight.d) |
|  | 6-gingerol | Acute/subacute toxicity | Mice | 250 mg/kg; Oral LD_50_ | 9.01 | （0.25÷9.01）× 60  ＝ 1.665 g/(60 kg.people body weight.d) |
|  | Freeze-dried ginger powder | Acute/subacute toxicity | Rats | - | - | - |
|  | Dry ginger decoction | Acute/subacute toxicity | Rats | 250 g/kg; Oral LD_50_ | 6.25 | （250÷6.25）× 60  ＝ 2400g/(60 kg.people body weight.d) |
|  | Roasted ginger decoction | Acute/subacute toxicity | Rats | 170.6 g/kg; Oral LD_50_ | 6.25 | （170.6÷6.25）× 60  ＝ 1637.76g/(60 kg.people body weight.d) |

The human dose was estimated by the equation: （X ÷ Y）× Z.

X: Dose use for one in Vivo study（X mg or g use in per kg animal body weight, usually noted as X mg(or g)/kg·Rat (or Mice).

Y: Convert factor to human.

Z: Human weight, usually fixed on 60 kg (60 kg for common adult’s body weight).

**Reference**

Additives, E.P.O., and Feed, P.O.S.U.I.A. (2015). Scientific Opinion on the safety and efficacy of glycyrrhizic acid ammoniated (chemical group 30, miscellaneous substances) when used as a flavouring for all animal species. *EFSA Journal* 13**,** 3971.

Ji, X., Huang, B., Wang, G., and Zhang, C. (2014). The ethnobotanical, phytochemical and pharmacological profile of the genus Pinellia. *Fitoterapia* 93**,** 1-17.

Li, P., Shen, J., Wang, Z., Liu, S., Liu, Q., Li, Y., He, C., and Xiao, P. (2020). Genus Paeonia: A comprehensive review on traditional uses, phytochemistry, pharmacological activities, clinical application, and toxicology. *Journal of Ethnopharmacology***,** 113708.

Li, X.-Q., Song, Y.-N., Wang, S.-J., Rahman, K., Zhu, J.-Y., and Zhang, H. (2018). Saikosaponins: a review of pharmacological effects. *Journal of Asian natural products research* 20**,** 399-411.

Mao, Q.-Q., Xu, X.-Y., Cao, S.-Y., Gan, R.-Y., Corke, H., and Li, H.-B. (2019). Bioactive compounds and bioactivities of ginger (Zingiber officinale Roscoe). *Foods* 8**,** 185.

Pastorino, G., Cornara, L., Soares, S., Rodrigues, F., and Oliveira, M.B.P. (2018). Liquorice (Glycyrrhiza glabra): A phytochemical and pharmacological review. *Phytotherapy Research* 32**,** 2323-2339.

Selyutina, O.Y., and Polyakov, N. (2019). Glycyrrhizic acid as a multifunctional drug carrier–From physicochemical properties to biomedical applications: A modern insight on the ancient drug. *International Journal of Pharmaceutics* 559**,** 271-279.

Stanisiere, J., Mousset, P.-Y., and Lafay, S. (2018). How safe is ginger rhizome for decreasing nausea and vomiting in women during early pregnancy? *Foods* 7**,** 50.

Tan, Y.-Q., Chen, H.-W., Li, J., and Wu, Q.-J. (2020). Efficacy, chemical constituents, and pharmacological actions of Radix Paeoniae rubra and Radix Paeoniae Alba. *Frontiers in Pharmacology* 11**,** 1054.

Tian, T., Chen, H., and Zhao, Y.-Y. (2014). Traditional uses, phytochemistry, pharmacology, toxicology and quality control of Alisma orientale (Sam.) Juzep: a review. *Journal of Ethnopharmacology* 158**,** 373-387.

Wang, J., Wang, L., Lou, G.-H., Zeng, H.-R., Hu, J., Huang, Q.-W., Peng, W., and Yang, X.-B. (2019). Coptidis Rhizoma: a comprehensive review of its traditional uses, botany, phytochemistry, pharmacology and toxicology. *Pharmaceutical Biology* 57**,** 193-225.

Xie, X., Chang, X., Chen, L., Huang, K., Huang, J., Wang, S., Shen, X., Liu, P., and Huang, H. (2013). Berberine ameliorates experimental diabetes-induced renal inflammation and fibronectin by inhibiting the activation of RhoA/ROCK signaling. *Molecular and Cellular Endocrinology* 381**,** 56-65.

Yang, X.-Z., and Wei, W. (2020). CP-25, a compound derived from paeoniflorin: research advance on its pharmacological actions and mechanisms in the treatment of inflammation and immune diseases. *Acta Pharmacologica Sinica* 41**,** 1387-1394.

Yao, R.-Y., Zou, Y.-F., and Chen, X.-F. (2013). Traditional Use, Pharmacology, Toxicology, and Quality Control of Species in Genus Bupleurum L. *Chinese Herbal Medicines* 5**,** 245-255.

Zhang, L.L., Xu, W., Xu, Y.L., Chen, X., Huang, M., and Lu, J.J. (2017). Therapeutic potential of Rhizoma Alismatis: a review on ethnomedicinal application, phytochemistry, pharmacology, and toxicology. *Annals of the New York Academy of Sciences* 1401**,** 90-101.
